# 

**Published:** 1869-12

**Authors:** 


### Editorial.

### CLOSE OF THE VOLUME.

This number closes the twenty-third volume of the DENTAL REGISTER. We have now but little disposition to retrospect further than to say that we think its pages will show a faithful record, to a considerable extent at least, of the progress of the Dental Profession for the past year. Observation will demonstrate the fact that definite onward movement is every year made by our profession. The faithful journalist should endeavor, with truth and good faith, to record the successive steps of this progress. But our country is so extended, and the members of the profession so active, and the spirit of investigation, invention and improvement so rife, that it is not possible that one observer however industrious, should be cognizant of all that occurs; but the great leading matters should not be overlooked.

We acknowledge with thankfulness the great encouragment and appreciation we have received in our editorial work during the past year, yet there have been some things that caused sadness and pain, prominent among them are our short comings. These make us cautious in promising great things for the future. We are fully warranted in the hope that we shall have a fuller and stronger co-operation of our professional brethren during the coming year than we have had in the past; we have no complaint for the past, but bright hopes for the future. T.

### SPECIAL NOTICES.

THE SEVENTH ANNUAL SESSION of the Iowa State Dental Society will be held at Muscatine, Iowa, commencing Tuesday evening, July 20, 1869, at 7 o'clock P. M., and continuing two whole days. We cordially invite all our professional brethren to be present, as we anticipate a good social reunion. The following gentlemen have been appointed to prepare and read essays on the occasion :

Dr. L. C. Ingersoll, "Specialties in Medicine and Dentistry ;" Dr. J. F. Sanborn, "Odontalgia ;" Dr. W. O. Kulp, "Notes on daily practice of Dental Surgery ;" Dr. I. P. Wilson, "Dentistry —Its Progress ;" Dr. P. T. Smith, "Dental Therapeutics ;" Dr. L. M. Jackson, "Dental Fees ;" Dr. J. M. Blakesley, "Defective Growth and Early Decay of Deciduous Teeth."

Subjects for Discussion—"Treatment for Exposed Dental Pulps," "Causes of Irregularity and best Mode of Treatment," "The necessity of Legislation to suppress Dental Quackery," "Anæsthetics in Dentistry," "Filling Teeth," "Mechanical Dentistry."

Any one having specimens of malformation, or cases of special interest to the profession, is requested to report the same. We would urge upon all Dentists the importance of being present, if possible, as it is a duty we owe ourselves, the public and the profession, and a pleasant and profitable session is anticipated. Dentists from neighboring States are cordially invited to meet with us and take part in the discussion.

L. C. INGERSOLL, *President.*

N. H. TULLOSS, *Cor. Secretary.*

---

MESSRS. GEORGE WATT & CO., DENTAL DEPOT, No. 9 Carlisle Building, south-west corner of Fourth and Walnut Streets, are constantly adding to their large and carefully selected stock of Dental goods. Having the BEST LIGHT IN THE CITY, by which to select teeth, (a very important consideration) being *practical Dentists of long experience*, and having a thorough and practical knowledge of all the drugs, medicines, chemicals and metals used in Dentistry, members of the profession who want life-like teeth, pure medicines, first-class instruments, carefully selected foils, properly purified or alloyed plate, or accurately tested chemicals, will be at no loss where to call. With pleasant rooms, competent and polite clerks, a thorough book-keeper, (recently added to our force) well selected stock, a good supply of patience, an overflow of kindness, a determination to please, and with *no other business to distract our attention*, we confidently invite our professional brethren to look to their own interests by giving us their patronage. Goods sold at manufacturers' prices.

### NEW AMALGAM

### A BEAUTIFUL AND EXCELLENT PREPARATION FOR FILLING TEETH.

***No. 1 Fine, No. 2 Medium Coarse, put up in 1-2 and 1 ounce packages, Circular enclosed.***

RETAIL PRICE, - - - $4.00 PER OUNCE.

A liberal discount to dealers. Manufactured by Dr. B. F. ARRINGTON, for

S. S. WHITE,

And sold at his Depots.

---

### PHILADELPHIA DENTAL COLLEGE.

108 North Tenth St., above Arch.

SEVENTH ANNUAL SESSION, 1869-70.

### FACULTY.

C. A. KINGSBURY, M.D., D.D.S.,
Professor of Operative Dentistry and Dental Histology.
J. H. McQUILLEN, M.D., D.D.S., Professor of Physiology.
ALBERT R. LEEDS, A. M., Professor of Chemistry.
WM. C. HEAD, D.D.S., Demonstrator of Operative Dentistry.
D. D. SMITH, D.D.S., Professor of Mechanical Dentistry and Metallurgy.
J. FOSTER FLAGG, D.D.S., Professor of Dental Pathology & Therapeutics.
HARRISON ALLEN, M.D., Professor of Anatomy and Surgery
WM. P. HENRY, D.D.S., Demonstrator of Mechanical Dentistry.

The Dispensary and Laboratory of the College will be open and preliminary lectures delivered by one of the Professors, every day during the latter half of October, commencing on the 19th inst. The regular course of instruction will commence on the 1st of November, and continue until the close of the ensuing February. Every opportunity will be afforded in the Clinic and Laboratory for obtaining a *practical* knowledge of Operative and Mechanical Dentistry.

### FEES.

| | |
|---|---|
| Matriculation (paid but once) - - - - - | $5 00 |
| Tickets for the *entire* Course, including the Demonstrators' | 100 00 |
| Diploma - - - - - - - - - - | 30 00 |

For further particulars, address J. H. McQUILLEN, DEAN,
No. 1112 Arch Street, Philadelphia.

### BALTIMORE COLLEGE OF DENTAL SURGERY

THIRTIETH ANNUAL SESSION, 1869–70.

### FACULTY.

THOMAS E. BOND, A.M., M.D., Prof. of Pathology and Therapeutics.
PHILIP H. AUSTEN, A.M., M.D., D.D.S., Prof. of Dental Science and Mechanism.
FERDINAND J. S. GORGAS, A.M., M.D., D.D.S., Prof, of Dental Surgery
A. SNOWDEN PIGGOT, A.M., M.D., Prof. of Chemistry and Metallurgy.
RUSSELL MURDOCH, A.M., M.D., Prof. of Anatomy.
HENRY REGINALD NOEL, A. M., M. D., Professor of Physiology.
HUGH McGINNIS GRANT, D.D.S., Demonstrator of Operative Dentistry.
THOMAS SOLLERS WATERS, D.D.S., Demonstrator of Mechanical Dentistry.
CLAUDE BAXLEY, M. D., Demonstrator of Anatomy.

The *Thirtieth* Annual Session will commence on the fifteenth of October, 1869, and continue until March, 1870.

| | |
|---|---|
| Lecture and Demonstration Fees, - - - | $115 |
| Matriculation Fee (paid only once) - - - - | 5 |
| Diploma Fee. - - - - - - - - | 30 |

Graduates of the Baltimore College of Dental Surgery can graduate at the Washington University of Medicine, Baltimore, on attending *one course* of Medical Lectures.

For information, address

F. J. S. GORGAS, M.D., Dean of the Faculty.

March. 8'6 *No.* 43 *Hanover St.*, *Baltimore*, *Md.*

### OHIO COLLEGE

OF

### DENTAL SURGERY.

The Twenty-fourth Annual Session of the Ohio College of Dental Surgery will commence on Tuesday the 15th of October, and continue five months.

### FACULTY.

JAMES TAYLOR, M. D., D. D. S.,
Emeritus Professor of Institutes of Dental Science.

C. KEARNS, M. D.,
Professor of Anatomy.

—— ——
Professor of Pathology.

GEORGE WATT, M. D., D. D. S.,
Professor of Chemistry and Therapeutics.

EDWARD RIVES, M. D.,
Professor of Physiology and Histology.

J. TAFT, D. D. S.,
Professor of Operative Dentistry and Dental Hygiene

H. R. SMITH, M. D., D. D. S.,
Professor of Mechanical Dentistry and Metallurgy,

J. A. WATLING, D. D. S.,

Professor of Clinical Dentistry.

WILL TAFT, D. D. S.
Demonstrator of Anatomy.

### TEXT BOOKS.

Gray's and Wilson's Anatomy; Dalton's or Carpenter's Physiology; Williams' Pathology; Fown's, Turner's, Graham's, or Brandt and Taylor's Chemistry; Taft's Operative Dentistry; Richardson's Mechanical Dentistry; Harris' Principles and Practice of Dental Science, Tomes' Dental Surgery, Watts' Chemical Essays, Harris' Dental Dictionary.

### TERMS OF ADMISSION.

Tickets must be procured at the beginning of the session.

| | |
|---|---|
| Tickets for the course, | $100 00 |
| Demonstrator's Ticket for Anatomy, | 5 00 |
| Matriculation Fee, but once, | 5 00 |
| Diploma Fee, | 30 00 |

### TERMS OF GRADUATION.

Two courses, (including one of dissections,) with a satisfactory dental pupilage.

Eight years reputable practice, or a satisfactory examination upon the branches of the Junior Course in this Institution will be received as equal to one course.

An acceptable thesis upon some subject on Dental science.

Must possess a good moral character, and be twenty-one years old.

For further information, address J. TAFT, *Dean.*
117 West Fourth Street

### The Dental Register

### OF THE WEST,

*Issued Monthly, at $3 a Year in Advance.*

DEVOTED TO THE INTERESTS OF THE DENTAL PROFESSION.

EDITED BY J. TAFT AND G. WATT.

The volume commences in January and closes in December.

The Register being issued monthly is always fresh, therefore indispensable to the practitioner who desires to keep posted up with the new inventions and improvements which are daily developed. Neither expense nor effort will be spared to give value and variety to its contents. Articles will be illustrated with well executed engravings whenever they will add to the interest or assist in explanation.

Its extensive circulation and frequency of issue makes it one of the BEST DENTAL ADVERTISING MEDIUMS in the United States.

RATES OF ADVERTISING.

One page, 1 year, $50. Half page, 1 year, $30. Quarter page, or card, 1 year $20.

All advertising bills payable in advance, or at furthest after the issue of the first number containing the advertisement. All transient advertisements to be paid for in advance

☞ CONTRIBUTIONS SOLICITED.

Address, J. TAFT, or "DENTAL REGISTER," Cincinnati Ohio.

Business Communications to

J. TAFT, Publisher,
No. 117 West Fourth St.

